# Effects of Infant Formula Supplemented With Prebiotics and OPO on Infancy Fecal Microbiota: A Pilot Randomized Clinical Trial

**DOI:** 10.3389/fcimb.2021.650407

**Published:** 2021-03-29

**Authors:** Bingquan Zhu, Shuangshuang Zheng, Kexin Lin, Xin Xu, Lina Lv, Zhengyan Zhao, Jie Shao

**Affiliations:** Department of Child Health Care, Children’s Hospital, Zhejiang University School of Medicine, National Clinical Research Center for Child Health, Hangzhou, China

**Keywords:** infants, milk, human, prebiotics, diolein, gastrointestinal microbiome

## Abstract

Several lines of evidence suggest that the intestinal microbiota plays crucial roles in infant development, and that it is highly influenced by extrinsic and intrinsic factors. Prebiotic-containing infant formula may increase gastrointestinal tolerance and improve commensal microbiota composition. However, it remains unknown whether supplementation of milk-formulas with prebiotics and 1,3-olein-2-palmitin (OPO) can achieve feeding outcomes similar to those of breastfeeding. In the present study, we investigated the effects of two kinds of infant formula with different additives on the overall diversity and composition of the fecal microbiota, to determine which was closer to breastfeeding. A total of 108 infants were enrolled, including breastfeeding (n=59) and formula feeding group (n=49). The formula feeding infants were prospectively randomly divided into a standard formula group (n=18), and a supplemented formula group(n=31). The fecal samples were collected at 4 months after intervention. Fecal microbiota analysis targeting the V4 region of the 16S rRNA gene was performed using MiSeq sequencing. The overall bacterial diversity and composition, key functional bacteria, and predictive functional profiles in the two different formula groups were compared with breastfeeding group. We found that the alpha diversity of the gut microbiota was not significantly different between the OPO and breastfeeding groups with Chaos 1 index (p=0.346). The relative abundances of *Enhydrobacter* and *Akkermansia* in the OPO group were more similar to those of the breastfeeding group than to those of the standard formula group. The gut microbiota metabolism function prediction analysis showed that the supplemented formula group was similar to the breastfeeding group in terms of ureolysis (p=0.297). These findings suggest that, when formula supplemented with prebiotics and OPO was given, the overall bacterial diversity and parts of the composition of the fecal microbiota would be similar to that of breastfeeding infants.

## Introduction

Breastfeeding is associated with several health benefits in children, including reduced risk of acute otitis media, gastrointestinal and other infections, atopic dermatitis, and asthma (AAP, 2012; Chantry et al., 2015; Fabrizio et al., 2018; WHO, 2018). Infant formula is an alternative to human milk if the mother is unable to breastfeed. Currently, many varieties of infant formula are available. The appropriate infant formula is chosen depending on the specific needs of the infant (Kleinman and Greer, 2014).

It has been supported by some evidences that breastfeeding is beneficial for colonization in human guts after birth (Johnson and Versalovic, 2012; Tanaka and Nakayama, 2017). While, the composition and function of intestinal microbiota play vital roles in development of digestion, metabolism, and activation of the immune system in infants, all of which impact later stages of life (Johnson and Versalovic, 2012; Tanaka and Nakayama, 2017). There is no conclusive evidence to confirm when and how the human gut flora starts to colonize, but it colonizes rapidly after birth and is influenced by a variety of factors (Collado et al., 2016). Feeding type (breastfeeding vs. formula) may have significant effect on gut microbiota shaping (Guaraldi and Salvatori, 2012). In addition to nutrients, human milk contains growth factors, cytokines, immunoglobulins, and digestion enzymes, and these factors influence the proliferation of several bacterial species (Roncada et al., 2012). Breastfed infants showed more stable microbiota than did formula-fed infants (Bezirtzoglou et al., 2011); consumption of even small amounts of infant formula resulted in a wider microbiota spectrum (Guaraldi and Salvatori, 2012). The levels of *Bifidobacterium* and *Lactobacillus* species in the intestinal microbiota of breastfed infants were relatively high in the first few months after birth. The intestinal microbiota composition of formula-fed infants was similar to that of older children. *Bacteroides*, *Clostridium*, and *Enterobacteriaceae* constituted the intestinal microbiota in formula-fed infants (Kunz et al., 2000). Apart from maternal impossibility or unwillingness to breastfeed, some infants might require infant formula for health reasons. Therefore, efforts were made to mimic the properties of human milk by adding probiotics and prebiotics in infant formula to improve microbiota maturation, as well as to ensure proper development and functioning of the immune system (Guaraldi and Salvatori, 2012).

Prebiotics are the third most prevalent component of human milk; they are almost absent in cow milk and infant formula (Kunz et al., 2000). They are non-digestible glycan compounds that stimulate the growth and activity of the intestinal microbiome (Johnson and Versalovic, 2012). Feeding infant formula supplemented with prebiotics led to a more adult-like microbiota diversity, alleviated atopic eczema, and decreased the occurrence of inflammatory bowel diseases in adulthood (Borewicz et al., 2019). Prebiotics such as galacto-OS, fructo-OS, and polydextrose can be added to the infant formula to improve its effects on the digestive system and immunity (Kolida and Gibson, 2007; Roberfroid et al., 2010; Sangwan et al., 2011; Miqdady et al., 2020). Studies have shown that these prebiotics stimulated the growth of specific strains of *Bifidobacterium* (also dominant in the gut of breastfed infants) and *Lactobacillus* and reduced growth of *Escherichia coli*, *Clostridium*, and other bacteria. The addition of these prebiotics resulted in colonic metabolic activity similar to that of the gut microbiota of infants who were fed human milk (Boehm et al., 2002; Knol et al., 2005; Kapiki et al., 2007; Costalos et al., 2008; Holscher et al., 2012; Srinivasjois et al., 2013). The most abundant triacylglycerol in human milk is 1,3-olein-2-palmitin (OPO) (Zou et al., 2013; Giuffrida et al., 2018), which can be synthesized from palm oil for supplementation to infant formula (Chen et al., 2004). Infants who were fed OPO had greater amounts of *Lactobacillus* and *Bifidobacterium* (Yaron et al., 2013).

Savino et al. (2003) showed that infant formula containing prebiotics and OPO decreased the frequency of colic. Other studies showed that supplementing prebiotics and OPO to infants in sufficient amounts softened the stool and increased the stool frequency but without diarrhea, and increased the ratio of *Bifidobacterium* to total stool bacteria (Schmelzle et al., 2003; Sherman et al., 2009; Closa-Monasterolo et al., 2017). Nevertheless, the effects of prebiotics and OPO in infant formula to achieve feeding outcomes similar to those associated with human milk remain unknown. Therefore, this study aimed to determine whether the gut microbiota composition in infants fed infant formula containing OPO and other prebiotics was similar to that of breastfed infants. The results in terms of different feeding methods and related intestinal microbiota might help in promoting better growth of infants and young children.

## Materials and Methods

### Study Design and Subject Selection

This was a multicenter, double-blind, randomized controlled trial. The infants were recruited between August 2015 and June 2017 at the Xihu District Maternal and Child Health Hospital, Jianggan District Maternal and Child Health Hospital, Lin’an District Maternal and Child Health Hospital, and Yuhang District Maternal and Child Health Hospital (Zhejiang, China). The inclusion criteria were: 1) ≤1 month of age; 2) singleton; 3) full-term (gestational age ≥37 weeks and <42 weeks); 4) birth weight: 2.5–4 kg; 5) either exclusively breastfed or the amount of formula feeding was >80% of the total intake within 72 h before enrollment; 6) no congenital anomalies, birth defects, genetic metabolic diseases, or chromosomal diseases; 7) no family history of genetic metabolic diseases; 8) no history of infectious diseases; 9) no antibiotics had been used since birth; and 10) the legal guardians of the infants agreed to participate in this clinical trial and provided written informed consent. The exclusion criteria were as follows: 1) either parent had a history of severe allergy; 2) diseases or high-risk factors that affected the growth and development of the infants, e.g., 5-min Apgar score <8, neonatal hypoxic-ischemic encephalopathy, intracranial hemorrhage, purulent meningitis, intrauterine infection, neonatal convulsions, hypoglycemia, and hospital stay >2 weeks in the neonatal period, among others; 3) other severe primary diseases of the cardiopulmonary, digestive, nerve, kidney, or circulatory systems; 4) participation in other clinical trials in the previous month; 5) use of antibiotics, probiotics, prebiotics, or synbiotics in the previous month; or 6) the investigators considered the newborn was inappropriate to participate in this clinical trial for any other reasons. Withdrawal criteria were as follows: 1) the infant was obviously intolerant to formula milk; or 2) the infant was treated with antibiotics, probiotics, prebiotics, or synbiotics during the trial. The study was approved by the ethics committee of all participating hospitals. The ethical approval was granted by the Ethics Committee of the Children’s Hospital, Zhejiang University School of Medicine (2015-IRB-035). Informed written consent was obtained from all parents or guardians before enrollment. The study was registered in the Resman Research Manager with the number ChiCTR2000039422.

### Intervention

All infants were enrolled at 1 month of age when they had their first health care check after birth in the maternal and child health hospital. The infants who were exclusively breastfed within 72 h before enrollment were assigned to the breastfeeding group. The infants whose intake of formula milk was >80% of the total milk intake within 72 h before enrollment were assigned to the standard formula group or the supplemented formula group using a random number table method. Both formulas were produced by Beingmate Baby & Child Food Co., Ltd. (Zhejiang, China). When leaving the factory, the formulas were deidentified. The outer packaging was the same, except that the labels ‘a’ and ‘b’ were affixed to the bottom of the bottle. The person in charge of the project team at each site randomly assigned each patient to bottles ‘a’ and ‘b’ using Excel. The staff at each project site matched the project number according to the time sequence of the baby’s entry into the group and distributed the milk powder according to randomization. The supplemented formula included OPO 4.0 g/100 g, fructooligosaccharide 0.8 g/100 g, and galactooligosaccharide 0.6 g/100 g, all other ingredients were the same in the two formulas. No probiotics and other prebiotics were included in two formula. For infants in the standard or supplemented formula groups, guardians and the researchers were all blinded to the grouping.

### Collection of Infant Information and Feces Collection

Demographic data and stool samples were collected. Breastfeeding was continued, or the infant formula specified in this trial was used for feeding. Follow-up visits to the outpatient clinic were conducted every 1 month to record feeding, defecation, crying, and spitting. If the baby cried for more than 5 minutes at a time and was difficult to comfort, and the reasons such as hunger, urination and drowsiness were excluded, one time of crying was recorded. Stool colorimetric card was used to help parents to judge the color of infant’s stool which including six degree as light yellow, golden yellow, green, brown, dark and white. Fecal samples were collected at 4 months of age. Follow-up ended in September 2017. The feces were collected during the outpatient follow-up using anal swabs (Zhang et al., 2018). The samples were stored at –80°C immediately after collection. If the parents refused to provide the anal swabs, they collected the stool at home and put it in a freezing tube, which was stored at –20°C, and the samples were transferred to the hospital in an icebox within 3 days. All samples in the hospital were stored under –80°C before further analysis.

### DNA Isolation, Amplicon Library Construction and Sequencing

DNA extraction was performed using the SDS lysate freeze-thaw method. Fecal bacterial genomic DNA was extracted using the PowerMax extraction kit (MoBio Laboratories, Carlsbad, CA), according to the manufacturer’s instructions, and stored at –80°C until further use. The amount of bacterial genomic DNA was analyzed using a NanoDrop ND-1000 spectrophotometer (Thermo Fisher Scientific, Waltham, MA, USA). The V4 region of bacterial 16 S rRNA gene was amplified by PCR using forward primer 515F (5’-GTGCCAGCMGCCGCGGTAA-3’) and reverse primer 806R (5’-GGACTACHVGGGTWTCTAAT-3’). PCR products were purified using AMPure XP Beads (Beckman Coulter, Indianapolis, IN, USA) and were quantized using a PicoGreen dsDNA, Assay Kit (Invitrogen, Carlsbad, CA, USA). The purified amplicons were then pooled in equimolar concentrations, and the final concentration of the library was determined using Qubit (Invitrogen). After quantification, an Illumina HiSeq 4000 pair-end 2 × 150 bp platform was used for sequencing.

### Bioinformatic Analysis

After sequencing, QIIME (version 1.9) was used for the quality control of FASTQ data. The reads were removed according to the following criteria: sequences <150 bp, average quality value less than 20, sequences with ambiguous bases, and single-nucleotide repeat sequences containing >8 bp. After removing the chimera sequences, clean reads were obtained. UCLUST was used for clustering the clean reads into 16S rRNA operational taxonomic units (OTUs) with a 97% similarity. The OTUs were annotated based on the SILVA128 database through VSEARCH (version 2.4.4). The alpha-diversity metrics and the beta-diversity metrics were performed by R package. The linear discriminant analysis (LDA) effect size (LEfSe) method was used for the specific characterization of the gut microbiota. The alpha parameter of LEfSe was set to 0.05, and the threshold on the logarithmic score of LDA analysis was set to 2.0. The prediction of metabolic function of gut microbiota was based on 16S rRNA maker gene sequences and analyzed using Faprotax 1.1. (http://www.zoology.ubc.ca/FAPROTAX).

### Statistical Analysis

Statistical analyses were performed using SAS 9.2 (SAS Institute, Cary, NY, USA). Continuous variables with normal distributions were expressed as means ± standard deviations (SD) and were compared using one-way analysis of variance (ANOVA). Non-normally distributed continuous variables were expressed as medians (interquartile range, IQR) and were analyzed using non-parametric tests. Categorical variables were expressed as numbers (percentages) and were analyzed using the chi-square test. A principal component analysis was performed to examine the differences in OTUs among the three groups. To determine the growth of the infants, the z-scores were calculated as (actual weight-standard weight)/standard deviation according to the WHO reference standard for children below two years old.

### Accession Number

The sequence data from this study were deposited in the GenBank Sequence Read Archive under accession number SRP (298825).

## Results

### Infant Characteristics

**Figure 1:**
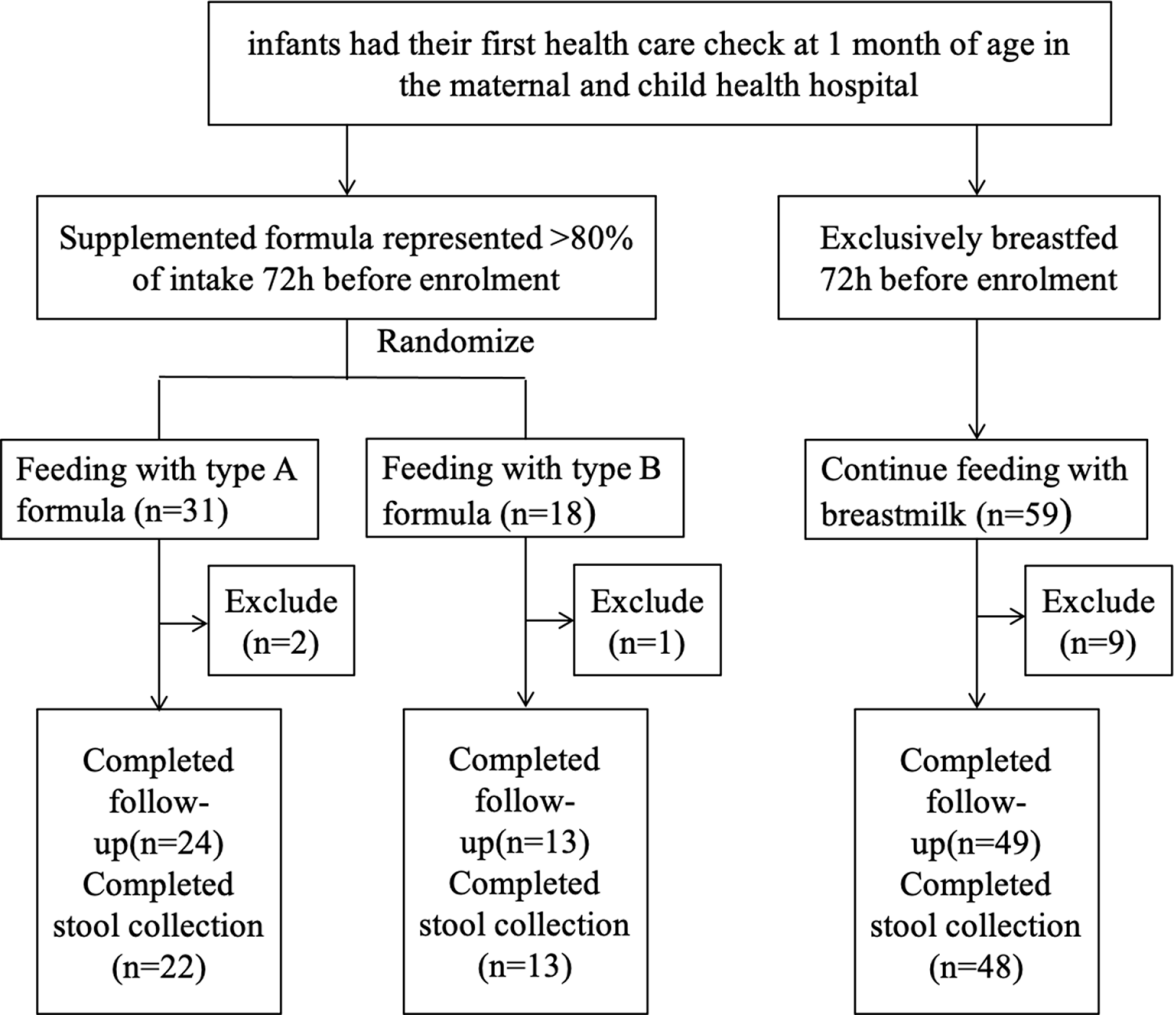
Study flowchart.

We prospectively enrolled a total of 108 infants; 59 infants were assigned to the breastfeeding group, and 49 infants were assigned to the supplemented formula group (n=31) or the standard formula group (n=18). Twelve infants were excluded due to antibiotic use, probiotic use, or being premature, with nine in the breastfeeding group, two in the supplemented formula group, and one in the standard formula group. There were five, four, and one were lost to follow-up in the supplemented formula, standard formula, and breastfeeding groups, respectively. Finally, 86 participants completed the trial (24, 13, and 49 in the supplemented formula, standard formula, and breastfeeding groups, respectively) (**Figure 1**). **Table 1** displays their characteristics. Parity was higher in the supplemented formula and standard formula groups (P=0.006), the father’s and mother’s educational levels were higher in the breastfeeding group (P<0.001), and family income was higher in the breastfeeding group (P=0.020). **Table 2** shows that there were no differences in weight-for-age (p=0.421), height-for-age (p=0.913), weight-for-height (p=0.258), or head circumference-for-age (p=0.109) among the three groups at 1, 2, 3 and 4 months of age. Significant difference had been found between three groups for stool characteristics and the color of stool at 4 months of age (P=0.017 and P=0.017). Infants fed with breastmilk had higher rate of shapeless stool and golden yellow stool comparing with formula groups. There was no significant difference between three groups for cry, occurrence of spitting and daily frequency of stool (P=0.260, P=0.937 and P=0.674) (**Table 3**).

**Table 1:** Baseline characteristics of the participants.

Characteristics	Supplemented formula group (n=24)	Standard formula group (n=13)	Breastfeeding group (n=49)	*P* value
Enrolled	31	18	59	
Followed up	24 (77.4%)	13(72.2%)	49 (83.1%)	0.569
Sex (male)	9 (37.5%)	7 (53.8%)	23 (46.9%)	0.599
Birth weight (kg)	3.27 ± 0.46	3.33 ± 0.42	3.34 ± 0.38	0.788
Mother’s age (years)	31.5 ± 5.0	32.2 ± 5.0	30.0 ± 4.2	0.194
Delivery mode				0.465
Normal labor	12 (50.0%)	4 (30.8%)	24 (49.0%)	
Cesarean section	12 (50.0%)	9 (69.2%)	25 (51.0%)	
Parity				0.006
1	8 (33.3%)	4 (30.8%)	33 (67.3%)	
≥2	16 (66.7%)	9 (69.2%)	16 (32.7%)	
Father’s educational level				<0.001
High school and below	20 (83.3%)	9 (69.2%)	33 (67.3%)	
University level and above	4 (16.7%)	4 (30.8%)	16 (32.7%)	
Mother’s educational level				<0.001
High school and below	20 (83.3%)	10 (76.9%)	11 (22.4%)	
University level and above	4 (16.7%)	3 (23.1%)	38 (77.6%)	
Monthly household income				0.020
≤10,000 yuan	17 (70.8%)	11 (84.6%)	23 (46.9%)	
> 10,000 yuan	7 (29.2%)	2 (15.4%)	26 (53.1%)	

**Table 2:** Growth conditions of the participants.

Age (months)	Index	Weight-for-age	Height-for-age	Weight-for-height	Head circumference-for-age
1	Supplemented formula group	-0.18 ± 0.76	-0.13 ± 0.96	-0.08 ± 0.83	-0.08 ± 1.13
	Standard formula group	-0.07 ± 0.90	0.26 ± 1.14	-0.41 ± 1.04	0.15 ± 0.81
	Breastfeeding group	0.24 ± 0.82	0.28 ± 0.88	-0.02 ± 1.02	-0.17 ± 0.76
	*P* value	0.108	0.203	0.452	0.480
2	Supplemented formula group	0.17 ± 0.89	0.17 ± 0.86	0.13 ± 1.05	-0.15 ± 1.01
	Standard formula group	0.39 ± 0.79	0.27 ± 0.84	0.32 ± 1.03	0.39 ± 0.85
	Breastfeeding group	0.55 ± 0.79	0.46 ± 0.82	0.33 ± 0.97	0.03 ± 0.77
	*P* value	0.190	0.345	0.721	0.189
3	Supplemented formula group	0.43 ± 0.92	0.13 ± 0.90	0.50 ± 1.26	0.02 ± 1.03
	Standard formula group	0.57 ± 0.84	0.48 ± 0.85	0.37 ± 1.22	0.26 ± 0.77
	Breastfeeding group	0.67 ± 0.88	0.60 ± 0.98	0.42 ± 0.80	0.12 ± 0.69
	*P* value	0.541	0.142	0.914	0.686
4	Supplemented formula group	0.55 ± 0.98	0.54 ± 0.85	0.34 ± 1.09	0.07 ± 1.03
	Standard formula group	0.99 ± 1.08	0.58 ± 0.96	0.88 ± 1.25	0.72 ± 0.98
	Breastfeeding group	0.72 ± 0.90	0.64 ± 1.04	0.50 ± 0.78	0.20 ± 0.81
	*P* value	0.421	0.913	0.258	0.109

**Table 3:** Changes in the characteristics of the stool.

Characteristics	Supplemented formula group (n=23)	Standard formula group (n=13)	Breastfeeding group (n=48)	P value
Stool form				**0.017**
Shapeless	0(0%)	1(7.7%)	12(25.0%)	
Shaped	23(100%)	12(92.3%)	36(75.0%)	
Daily frequency of stool				0.674
<1	7(30.4%)	4(30.8%)	14(29.2%)	
1~3	16(69.6%)	9(69.2%)	31(64.6%)	
>3	0(0%)	0(0%)	3(6.3%)	
Color of stool				0.017
Light Yellow	2(8.7%)	2(15.4%)	1(2.1%)	
Golden Yellow	17(73.9%)	8(61.5%)	46(95.8%)	
Green	4(17.4%)	3(23.1%)	1(2.1%)	
Cry				0.260
Yes	10(43.5%)	9(69.2%)	26(54.2%)	
No	13(56.5%)	4(30.8%)	22(45.8%)	
spitting				0.937
Yes	5(21.7%)	3(23.1%)	12(25.5%)	
No	18(78.3%)	10(76.9%)	35(74.5%)	

### Gut Microbial Diversity Analysis

**Figure 2:**
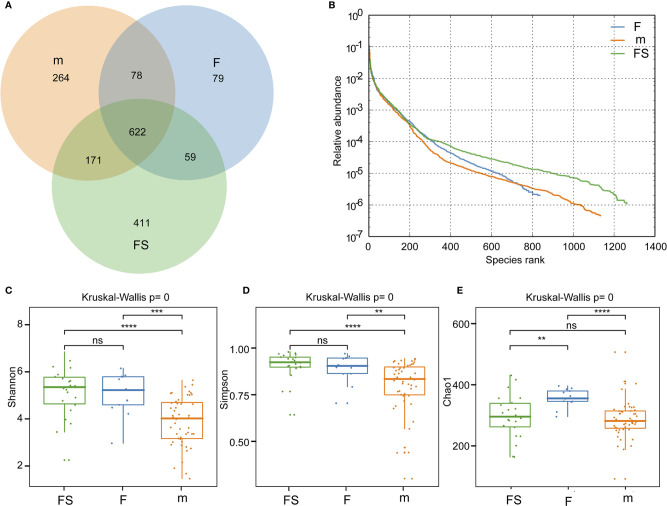
Analysis of gut microbial diversity. **(A)** Common and unique number of OUTs in the three groups. **(B)** Rank abundance curve of the three groups. **(C)** Community richness analysis by Chao 1 index. **(D)** Community diversity analysis by Shannon index. **(E)** Community diversity analysis by Simpson index. fs, supplemented formula group (n = 22); f, standard formula group (n = 13); m, breastfeeding group (n = 48). **(p < 0.01); ***(p < 0.001); ****(p < 0.0001); ns, no significant.

We analyzed bacterial diversity to determine the effects of supplemented formula and standard formula on the gut microbiota. A Venn diagram showed that, compared with standard formula, supplemented formula produced bacteria similar to those of the breastfeeding group (**Figure 2A**). The abundance grade curve showed that OTUs of the supplemented formula group and the breastfeeding group were both around 1200, and that the OTUs of the standard formula group were around 800, which shows the detection depth is sufficient in three groups. (**Figure 2B**). The mean Chaos 1 index showed that there were no significant differences between the supplemented formula group and the breastfeeding group (297.2 ± 71.9 vs. 286.8 ± 63.3, respectively, p=0.346), while there were significant differences between the standard formula group and the breastfeeding group (357.1 ± 29.6 vs. 286.8 ± 63.3, respectively, p=3.40×10^-5^). These findings suggest that the supplemented formula group was closer to the breastfeeding group in terms of gut microbial richness (**Figure 2C**). The Shannon and the Simpson indexes showed that both the supplemented formula group and the standard formula group had significant differences with the breastfeeding group in terms of diversity (**Figures 2D, E**).

### Gut Microbial Composition Analysis

**Figure 3:**
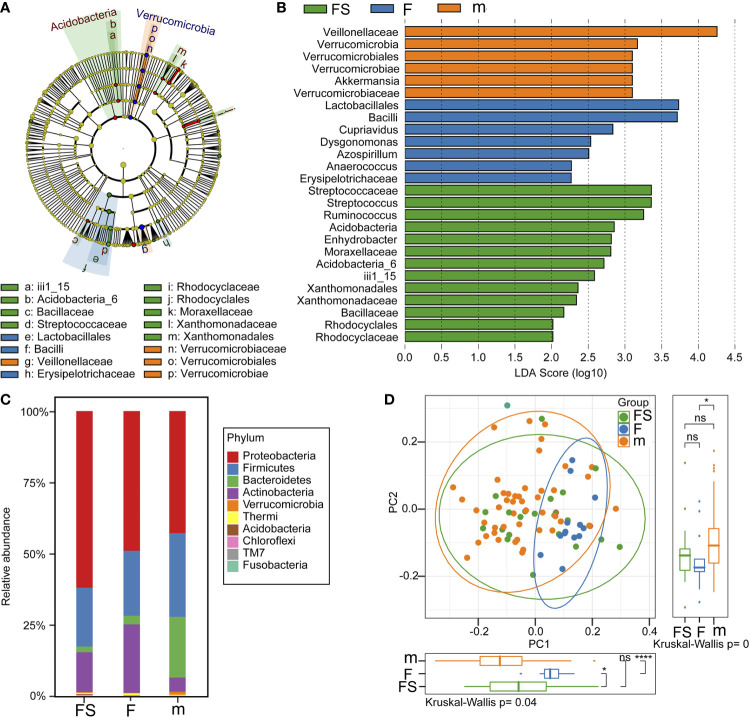
Analysis of gut microbial community structure. **(A)** A cladogram representation of data in the three groups. **(B)** LDA differential contribution analysis. **(C)** Species composition abundance in the three groups. **(D)** PCoA analysis. fs, supplemented formula group (n = 22); f, standard formula group (n = 13); m, breastfeeding group (n = 48). *(p < 0.05); ****(p < 0.0001); ns, no significant.

We analyzed the community structure to assess alterations due to supplemented formula or standard formula in the fecal microbiota. The compositions of the fecal microbiota in the two formulas were assessed at various taxonomic levels. Using the SILVA128 database, the sequences were classified as 20 phyla, 123 families, and 183 genera. LEfSe analysis identified 26 signature bacterial taxa that were differentially altered, with an LDA score >2, showing that there was significant structural difference among the three groups. There was a greater enrichment of veillonellaceae and verrucomicrobiae genus akkermansia in the breastfeeding group than in the formula feeding groups, of streptococcaceae genus streptococcus and ruminococcus in the supplemented formula group, but of vacilli genus lactobacilales in the standard formula group (**Figures 3A, B**). Generally, the gut microbiota was dominated by Proteobacteria, Firmicutes, Bacteroidetes, Actinobacteria, verrucomicrobia, in descending order. Notably, the abundant phylum, such as proteobacteria was enriched in the supplemented formula group (p=0.015), actinobacteria was enriched in the standard formula group (p=0.011), while verrucomicrobia were enriched in the breastfeeding group (p=0.0003) (**Figure 3C**). A principal component analysis for beta diversity showed that the intestinal microbial community structure in the supplemented formula group was closer to the breastfeeding group. The differences between the supplemented formula group and the breastfeeding group were not significant for PCoA1 (p=0.055) or PCoA2 (p=0.087), The differences between the standard formula group and the breastfeeding group were significant for PCoA1 (p<0.001) or PCoA2 (p=0.047), and the differences between the supplemented formula group and the standard formula group were also significant for PCoA1 (p=0.013) but not significant for PCoA2 (p=0.601) (**Figure 3D**).

**Figure 4:**
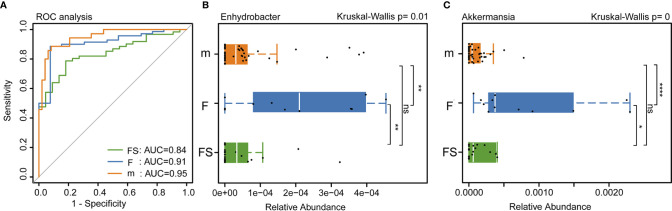
Analysis of intestinal bacteria that affect the health of newborns. **(A)** Accuracy of bacterial classification assessment by ROC curve. **(B)** Relative abundance of Enhydrobacter in three groups. **(C)** Relative abundance of Akkermansia in three groups. fs, supplemented formula group (n = 22); f, standard formula group (n = 13); m, breastfeeding group (n = 48). *(p < 0.05); **(p < 0.01); ****(p < 0.0001); ns, no significant.

We used bacterial markers to classify the species and receiver operating characteristic curves to determine classification accuracy. The areas under the curve of the supplemented formula group, the standard formula group, and the breastfeeding group were 0.84, 0.91 and 0.95, respectively. This suggests that the bacterial classification method was reliable (**Figure 4A**). We analyzed the bacteria that had been reported in the literature to affect the development of newborns and found that the relative abundance of *Enhydrobacter* showed no significant difference between the supplemented formula group and the breastfeeding group (p=0.324); however, the standard formula group was significantly different from the breastfeeding group (p=0.0063) (**Figure 4B**). The relative abundance of *Akkermansia* also showed no significant difference between the supplemented formula group and the breastfeeding group (p=0.374); however, in the standard formula group, abundance was significantly lower than in the breastfeeding group (p=1.40×10^-5^) (**Figure 4C**). These results suggest that the supplemented formula group was closer to the breastfeeding group in terms of specific strains that affect the development of newborns. Collectively, these changes in the fecal microbiota revealed that prebiotics and OPO might improve parts of the gut microbiota of formula-fed infants, bringing it closer to the effects of breast milk.

### Inferred Functional Changes Related to Infant Health

**Figure 5:**
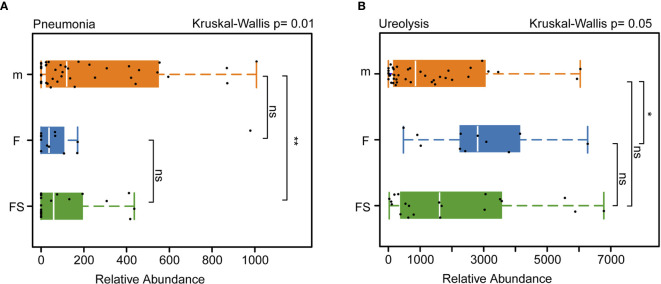
Analysis of the function of intestinal bacteria that affect newborns health. **(A)** Relative abundance of intestinal bacteria related to septicemia in three groups. **(B)** Relative abundance of intestinal bacteria related to ureolysis in three groups. fs, supplemented formula group (n = 22); f, standard formula group (n = 13); m, breastfeeding group (n = 48). *(p < 0.05); **(p < 0.01); ns, no significant.

We analyzed the function of the intestinal microbes related to the health of infants and found that, in terms of the abundance of bacteria associated with pneumonia, both the supplemented formula (p=0.0094) and the standard formula (p=0.0142) were different from the breastfeeding groups (**Figure 5A**). Interestingly, the supplemented formula group was very close to the breastfeeding group in terms of ureolysis (p=0.297) (**Figure 5B**). This suggests that prebiotics and OPO might improve parts of the function of the gut microbiota of formula-fed infants, bringing it closer to the effects of breast milk.

## Discussion

The colonization of intestinal microbiota in the early stages of human life plays a major role in the neurodevelopmental processes and growth of the infant. Studies have investigated the positive impacts of breastfeeding on the economy and on immunology and nutrition. The inverse association of sudden infant death syndrome, lower respiratory tract infections, otitis media, and gastroenteritis with breastfeeding is well known (Rouw et al., 2018). Elements such as genetic factors, age, delivery and feeding modes, and environment, affect the development of intestinal microbiota during infancy (Bäckhed et al., 2015).

Recently, studies have demonstrated the role of breastfeeding on the infant gut microbiota. A large multicenter study reported that breastfeeding was the most significant factor associated with microbiome structure in early life. Cessation of breastfeeding was shown to result in faster maturation of the gut microbiome, as marked by the presence of the phylum Firmicutes (Bäckhed et al., 2015). In the first month, the species *Bifidobacterium* dominates the intestinal microbiota of both breastfed and formula-fed infants. However, proportion of *Bifidobacterium* is higher in breastfed infants. Moreover, higher proportions of *Enterobacter*, *Bacteroides*, and *Clostridium* were found in formula-fed infants (Rautava et al., 2016).

Having recognized the importance of human breastmilk in shaping the gut microbiome, researchers have made numerous attempts to mimic the composition of human breast milk by supplementing infant milk formulations with live bacteria (probiotics), non-digestible fibers, nucleotides and oligosaccharides (prebiotics), and bovine lactoferrin with the aim of establishing gut microbiota in formula-fed infants (Fanaro et al., 2003; Singhal et al., 2008; Guaraldi and Salvatori, 2012). several studies reported benefits of feeding neonates milk fat globule membrane and probiotic-supplemented formula (He et al., 2019a; He et al., 2019b). Mah et al. attempted to administer a probiotic formula to Asian infants during the first 6 months of life and found that the supplemented strains colonized the gut but did not persist once administration ceased (Mah et al., 2007). Other researchers fed infants formula milk supplemented with prebiotics after the infants were treated with antibiotics, found that the prebiotic-supplemented formula increased fecal *Bifidobacterium* without producing any gastrointestinal symptoms (Brunser et al., 2006). Which emphasizing the need for supplemented formula milk that mimics human breast milk.

The most optimal doses of prebiotics and OPO in infant formula to achieve feeding outcomes similar to those of human milk remain unknown. In this study, we investigated the effect of infant formula supplemented with prebiotics and OPO on the composition of intestinal microbiota in infants and determined the type of supplement that would make infant formula more similar to human milk.

In our study, the number of bacterial species was found to be similar in the supplemented formula and breastfeeding groups and fewer in the standard formula group. The structure of the intestinal microbial community in the supplemented formula group was closer to that of the breastfeeding group. The number of aerobic species in the standard formula group was higher than those of the supplemented formula and breastfeeding groups. However, the number of Gram-negative species was the highest in the breastfeeding group and the lowest in the standard formula group; the number of Gram-positive species was the lowest in the breastfeeding group and the highest in the standard formula group. It is not clear whether these changes are beneficial to the infant or not as this question was beyond the scope of the present pilot study. We found no significant differences in the overall growth of the infants in the three groups; however, this study was conducted only up to the age of 4 months. Hence, future studies with a follow-up to the age of 12 months are recommended. Nevertheless, supplementation of prebiotics or probiotic strains showed conflicting results, as highlighted by Vandenplas and Savino (2019), probably because of the different underlying conditions, strains, and prebiotics tested (Wopereis et al., 2018; Vandenplas and Savino, 2019; Miqdady et al., 2020).

*Enhydrobacter* and *Akkermansia* play important roles in infant health and the development of immunity to diseases. *Enhydrobacter* is a gas-vacuolated, facultative anaerobic rod which is a major taxon in humans, found on human skin (Lee et al., 2012)and in the ocular microbiome. It is also found in high abundance in human colostrum, where it is involved in the use of the nutrients and immune development (Toscano et al., 2017).

*A. muciniphila *is a member of the phylum Verrucomicrobia. It was first isolated in 2004 in a study identifying new mucin-degrading bacteria from human feces. *Akkermansia* is considered a true symbiont for humans, and it has been found in the majority of tested individuals (Collado et al., 2007). It is one of the most frequent enterotypes in the human gut microbiome (Arumugam et al., 2011). Neonatal dysbiosis is characterized by a lower abundance of *Akkermansia*, and it has been associated with atopy and development of asthma (Fujimura et al., 2016).

In the present study, we found that the supplementation of infant formula with prebiotics and OPO increased amounts of *Enhydrobacter* and *Akkermansia* in the gut. Significant differences were observed in terms of relative abundance of *Enhydrobacter* in the standard formula group compared with the breastfeeding group. Similar results were observed in terms of the relative abundance of *Akkermansia*. These results suggest that supplementation of the infant formula induces changes in the microbiota compared with that of human milk.

Studies showed that prebiotics and OPO in sufficient amounts soften the stool in infants and increase the stool frequency, but without diarrhea (Schmelzle et al., 2003; Sherman et al., 2009). The infant formula used in the supplemented formula group and standard formula group improved the shapeless stool of infants but had lower occurrence of golden yellow stool. In addition, given the small sample size in this study, there were no significant differences in terms of defecation frequency and occurrence of spitting. The supplemented formula group had lower ratio of cry, but there were no significant differences between three groups. A larger sample-size study is needed for further exploration of these conditions.

The supplementation of prebiotics and OPO in infant formula promoted ureolysis. Human milk contains about 15% of its nitrogen in the form of urea, and an appropriate ureolytic capacity is likely necessary to meet the requirements of proper growth (Fuller and Reeds, 1998; Mora and Arioli, 2014). Specific strains of gut microbes possess ureolytic capacity and participate in nitrogen recycling for use by the host (LoCascio et al., 2010; Mora and Arioli, 2014). In the present study, the supplemented formula group exhibited intestinal microbial functions, especially ureolysis, similar to those of the breastfeeding group. There is crosstalk between the gut and the lung, the so-called gut-lung axis (Marsland et al., 2015; Bingula et al., 2017; Frati et al., 2018). Gut microbiota dysbiosis leads to dysregulation of the immune system that can affect the development of autoimmune diseases such as asthma, chronic pulmonary obstructive disease, cystic fibrosis, and lung cancer later in life (Chunxi et al., 2020). In particular, the gut-lung axis plays a major role in lung infection by distally driving responses to lung pathogens in pneumonia (Gray et al., 2017). An animal study showed that the depletion of gut microbiota favored the development of pneumonia and that fecal microbiota transplantation reversed enhanced host defenses against pneumonia (Schuijt et al., 2016). The presence of *Enterococcaceae* in the gut has been associated with the development of community-acquired pneumonia (Ren et al., 2020), and the use of probiotics contributes to protection against bacterial pneumonia (Vieira et al., 2016) and to recovery from viral respiratory infections (Waki et al., 2014). A study showed that gut *Proteobacteria* and *Firmicutes* dysbiosis is involved in lung inflammation and lower respiratory tract infections (Li et al., 2019). These findings suggest that promoting the gut microbiota in newborns using prebiotics might be conducive to reduce the occurrence of pneumonia.

This study has a few limitations. The sample size was small, and very few exclusively formula-fed infants were available. Most of the infants received mixed feeding, and parents held some misconceptions about the infant formula provided in this study; therefore, they were reluctant to participate in the study, making it difficult for the infant formula group to attain an adequate number of participants. Moreover, no block randomization was made at any center when recruiting the participants, resulting in differences in the number of participants between the supplemented formula and standard formula groups. Follow-up was conducted only for a limited age of the participants, and the effect of long-term consumption of infant formula was not explored.

In conclusion, the findings of this study suggest that the supplementation of prebiotics and OPO in infant formula shapes the structure and function of the gut microbiota. The overall diversity and composition of the intestinal microbiota in infants who were fed infant formula supplemented with prebiotics and OPO were closer to those of human milk than to those of standard formula.

## Data Availability Statement

The datasets presented in this study can be found in online repositories. The names of the repository/repositories and accession number(s) can be found below: https://www.ncbi.nlm.nih.gov/genbank/, SRP298825.

## Ethics Statement

The studies involving human participants were reviewed and approved by The Medical Ethical Committee of Children’s Hospital, Zhejiang University School of Medicine. Written informed consent to participate in this study was provided by the participants’ legal guardian/next of kin.

## Author Contributions

JS conducted and designed the research. BZ, SZ, KL, LL, and XX performed the experiments. SZ and KL analyzed the data. BZ, ZZ, and SZ wrote the paper and edited the manuscript. ZZ, BZ and JS interpreted the results. All authors read and approved the final manuscript.

## Funding

This study was supported by the National Key Research and Development Program of China (2019YFC0840702, J. Shao, Principal Investigator) and the National Natural Science Foundation of China (81773440, J. Shao, Principal Investigator). The content is solely the responsibility of the authors and does not necessarily represent the official views of the funding agencies. The standard and supplemented formulas were donated by Beingmate Co. Ltd. The funder was not involved in the study design, collection, analysis, interpretation of data, the writing of this article or the decision to submit it for publication.

## Conflict of Interest

The authors declare that the research was conducted in the absence of any commercial or financial relationships that could be construed as a potential conflict of interest.
